# Superficial basal cell carcinoma, think deeper: Step sectioning of skin biopsy specimens yields 14% more aggressive subtypes

**DOI:** 10.1371/journal.pone.0256149

**Published:** 2022-01-20

**Authors:** Mary-Ann El Sharouni, Paul J. van Diest, Willeke A. M. Blokx

**Affiliations:** 1 Department of Dermatology, University Medical Center Utrecht, University Utrecht, Utrecht, The Netherlands; 2 Department of Pathology, University Medical Center Utrecht, University Utrecht, University Utrecht, Utrecht, The Netherlands; Brown University Warren Alpert Medical School, UNITED STATES

## Abstract

**Introduction:**

Because of different therapeutic regimens for superficial and non-superficial basal cell carcinomas (BCCs), accurate histopathological examination of a punch biopsy to determine its subtype is essential. The aim of the current study was to evaluate the additional yield of a more extensive step-section method to that of a standard histologic examination at 4 levels.

**Material and methods:**

Data for this prospective study was obtained from the Pathology department of a Dutch tertiary hospital. Biopsy specimens of subsequent patients from March 2019 to June 2020 were sectioned to 8-levels instead of the regular 4-levels. Only patients with a superficial BCC subtype in the first 4-levels of sectioning were included (n = 100). After 8-level sectioning, it was recorded in which level (5–8) a more aggressive BCC component was found (i.e. nodular, infiltrative, or micronodular). Patients were followed-up to evaluate further treatment, and in case of excision, the excision specimen was reviewed to determine the BCC subtype. A logistic regression was performed to assess characteristics associated with a more aggressive BCC component in levels 5–8.

**Results:**

In 14 patients (14%) a more aggressive component was found in levels 5–8, all with a nodular component. Thirteen of these patients underwent excision, confirming a more aggressive BCC subtype. Of the 86 patients that had no deeper BCC component in levels 5–8, 26 (30.2%) underwent excision; In 4 patients no residual BCC was found, in 15 patients superficial BCC, and in 7 a more aggressive BCC subtype (1 nodular and 6 a combination of superficial/nodular/infiltrative). In multivariable analysis, head&neck localization was associated with finding a more aggressive BCC subtype in levels 5–8 (OR 6.41 (95%CI 1.56–26.30), p = 0.01)).

**Conclusions:**

More extensive sectioning of superficial BCC biopsy specimens, especially in the head&neck area, leads to a more accurate BCC subtype diagnosis requiring different clinical management strategies.

## Introduction

Basal cell carcinoma (BCC) is the most common form of all cancers in white populations [1–3]. Although mortality is very low (<0.1%) [4], it can cause considerable morbidity and places a heavy burden on public health costs [5,6]. Four main histological subtypes of BCC exist: superficial (sBCC) and non-superficial BCC, and the latter can be further categorized into nodular (nBCC), infiltrative (iBCC), and micronodular BCC (mBCC) [7]. For clinical management, the differentiation between sBCC, nBCC, and iBCC/mBCC is of importance because of different therapeutic regimens. It is therefore recommended to perform a punch biopsy of ambiguous lesions, large BCCs and BCCs located in high-risk areas, to determine its subtype [2]. sBCC can be treated with local therapy, such as cryotherapy, imiquimod, fluorouracil cream and photodynamic therapy, whereas the recommended treatment for non-sBCC subtypes is generally excision [2]. Misdiagnosis of sBCC and consequently topical treatment of underdiagnosed more aggressive BCC subtypes may lead to more recurrences. Therefore, accurate histopathological examination of a punch biopsy is essential. The aim of the current study was to evaluate the yield of an extensive 8 step-section method in patients who were initially diagnosed with sBCC based on a routine performed histologic examination at 4 levels.

## Patients and methods

### Collection of data

Data for this prospective institutional-based study were obtained from the University Medical Center (UMC) Utrecht, a tertiary hospital in the Netherlands. The Pathology department at the UMC Utrecht receives specimens from its tertiary Dermatology department, as well as from General Practitioners and non-academic Dermatology clinics. The need for ethics approval for this study was waived by the Biobank Research Ethics Committee.

### Study population

Between March 2019 and June 2020, biopsy specimens of 100 subsequent patients referred to the Pathology department of the UMC Utrecht, the Netherlands, and seen by a specialized dermatopathologist (WBL), were sectioned and hematoxylin-eosin (HE) stained to 8-levels instead of the regular 4-levels. Only patients with a sBCC in the first 4-levels of sectioning were included. For each patient, clinical and pathological variables were recorded, including date of diagnosis, age, sex, localization, clinical differential diagnosis and biopsy specimen size. The outcome of interest was “a more aggressive BCC subtype” in a deeper level (level 5–8) of sectioning, defined as nBCC, iBCC or mBCC. For each punch biopsy, the standard histologic examination started at a depth approximately 1000μm, and continued at an interval of 100μm, so that levels 1–4 were cut at 1000μm, 1100μm, 1200μm and 1300μm, respectively. At each level, 10 sections of 4μm were cut, of which the first section was hematoxylin-eosin stained and evaluated. The more extensive step-section method continued at 1500μm, so that levels 5–8 were cut at a depth of 1500μm, 1600μm, 1700μm and 1800μm, respectively (S1 Fig). After 8-level sectioning, it was recorded at which level a more aggressive BCC component was found, and the tumor thickness was measured. Tumor thickness was measured from the granular layer of the epidermis to the deepest tumor cell. sBCC subtype was defined as a tumor with a maximum tumor depth of ≤0.4mm and/or localization above the superficial vascular plexus [8]. Patients were followed-up to evaluate further treatment, and in case of excision, the excision specimen was reviewed to determine the BCC subtype.

### Statistical analysis

Categorical variables were summarized as numbers and percentages. Continuous variables were summarized as median with range for non-normally distributed data or mean with standard deviation (SD) for normally distributed data. Differences in proportions and medians were analyzed using chi-square tests or Mann-Whitney U test, respectively. Univariable and multivariable logistic regression analyses were performed to estimate odds ratio’s (ORs) and 95% confidence intervals (CIs) to evaluate which clinical variables (age, sex and localization) were associated with a more aggressive BCC component in the biopsy specimen. All analyses were performed using SPSS version 26.0. A two-sided p-value of <0.05 was considered statistically significant.

## Results

### Patients

Table 1 shows the clinicopathological characteristics of all 100 included patients, 55 males and 45 females. The median age was 59 years (range 28–85). The majority of BCCs were located on the head and neck (51%). Biopsies were taken with a 2, 3, 4 and 5mm punch biopsy in 30, 65, 4 and 1 patient(s), respectively. A total of 64 patients were referred by a non-academic dermatology clinic, 22 by an academic dermatologist and 14 by a general practitioner. The clinical diameter was available in 56 patients.

**Table 1:** Clinicopathological characteristics of all included patients.

	Total, n = 100
**Gender (n (%))**	
Female	45 (45.0)
Male	55 (55.0)
**Median age at diagnosis in years (range)**	59 (28–85)
**Localization (n (%))**	
Head and neck	11 (11.0)
Trunk	51 (51.0)
Arms	7 (7.0)
Legs	31 (31.0)
**Clinical diagnosis BCC (n (%))**	
No	11 (11.0)
Yes	89 (89.0)
**Median clinical diameter in cm (range)** *	0.8 (0.2–4.0)
**Size biopsy (n (%))**	
2mm	30 (30.0)
3mm	65 (65.0)
4mm	4 (4.0)
5mm	1 (1.0)
**Median max depth biopsy in mm (range)**	0.3 (0.2–1.1)

* Available for 56 patients.

### Deeper component and logistic regression

**Fig 1:**
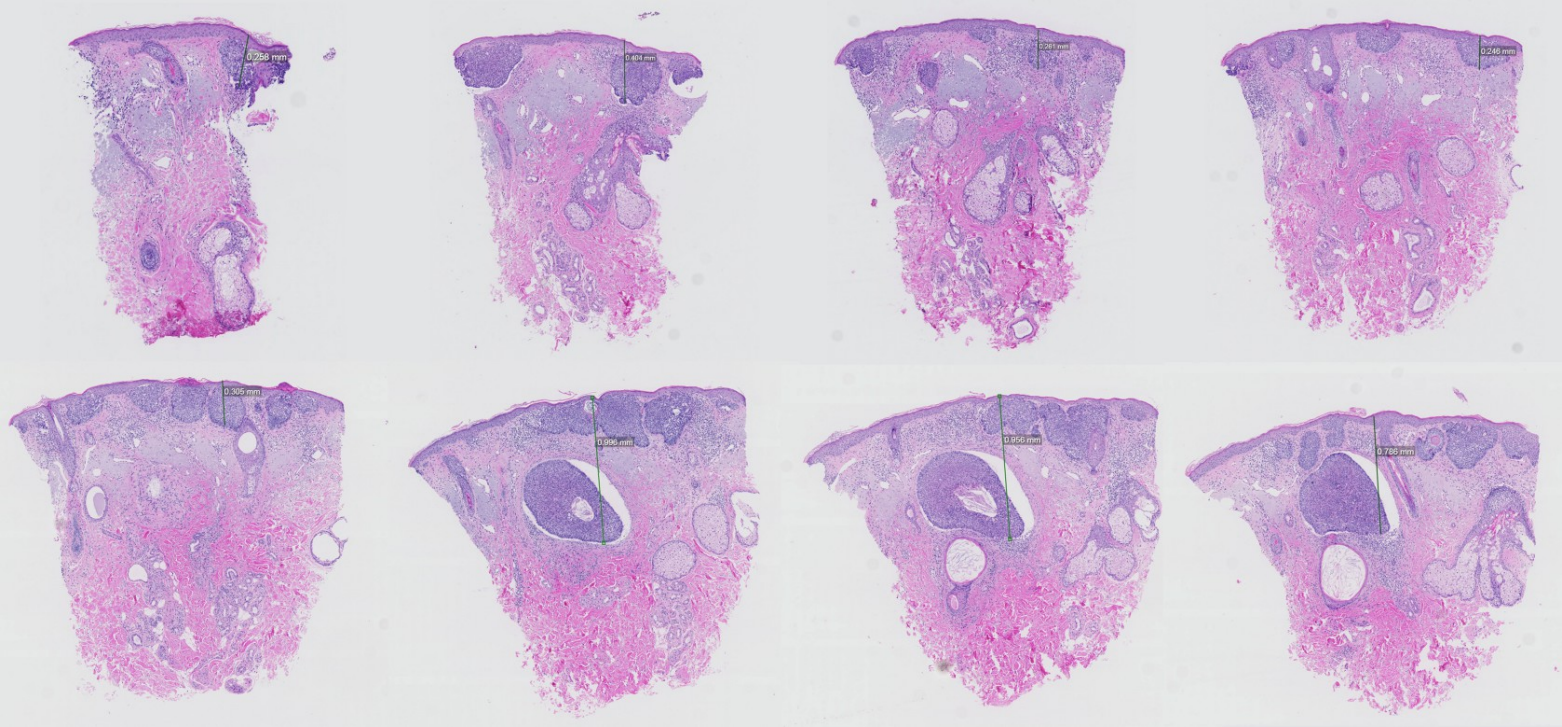
Examples of the HE sections of the standard histologic examination at 4 levels (HE 1–4, upper panel from left to right) and the HE sections of the 4 additional levels (HE 5–8, lower panel from left to right). In this example, an additional nodular component was found in level 6, 7 and 8.

In 14 patients (14%) a more aggressive component was found in levels 5–8; all with a nodular component. An example in shown in Fig 1. Table 2 compares clinicopathological characteristics of these 14 patients to patients with only sBCC in levels 5–8. Patients with a nodular component in levels 5–8 were more likely to have a BCC located in the head&neck region (35.7% vs. 7.0%, p = 0.02). Of the 14 patients with a nodular component in levels 5–8, 4 patients had their first nBCC component in slide 5, 7 patients in slide 6, 1 patient in slide 7 and 2 patients in slide 8 (Table 3). Upon multivariable analysis (included variables: sex, age at diagnosis and anatomic location), head&neck localization remained the only clinicopathological variable associated with finding a more aggressive subtype in deeper HE levels (OR 6.41 (95% CI 1.56–26.30), p = 0.01).

**Table 2:** Clinicopathological characteristics of patients with a more aggressive subtype in additional step sections (levels 5–8) vs. the remaining patients.

	More aggressive subtype in levels 5–8 N = 14	No aggressive subtype in levels 5–8 N = 86	p-value
**Gender (n (%))**			0.18
Female	4 (28.6)	41 (47.7)	
Male	10 (71.4)	45 (52.3)	
**Median age at diagnosis in years (range)**	67 (41–85)	58 (28–83)	0.30
**Localization (n (%))**			0.02
Head and neck	5 (35.7)	6 (7.0)	
Trunk	5 (35.7)	46 (53.6)	
Arms	1 (7.1)	6 (7.0)	
Legs	3 (21.4)	28 (32.6)	
**Median clinical diameter in cm (range)**	1.1 (0.3–2.1)*	0.8 (0.2–4.0) **	0.40
**Size biopsy (n (%))**			0.90
2mm	26 (30.2)	4 (28.6)	
3mm	56 (65.1)	9 (64.3)	
4mm	3 (3.5)	1 (7.1)	
5mm	1 (1.2)	(0.0)	
**Median max depth biopsy in mm (range)**	0.6 (0.5–1.1)	0.3 (0.2–0.4)	<0.0001

* Available in 11 patients.

** Available in 45 patients.

**Table 3:** Overview of HE levels of the 14 patients with a more aggressive BCC component in deeper HE slides (levels 5–8). All patients had a deeper, nodular component. sBCC = superficial basal cell carcinoma, nBCC = nodular basal cell carcinoma.

Patient nr	Level							
	Standard sectioning level				Additional, deeper sectioning levels			
	1	2	3	4	5	6	7	8
**1**	sBCC	sBCC	sBCC	sBCC	sBCC	nBCC	nBCC	nBCC
**2**	sBCC	sBCC	sBCC	sBCC	sBCC	nBCC	nBCC	nBCC
**3**	sBCC	sBCC	sBCC	sBCC	sBCC	nBCC	nBCC	NA
**4**	sBCC	sBCC	sBCC	sBCC	nBCC	nBCC	nBCC	NA
**5**	sBCC	sBCC	sBCC	sBCC	sBCC	nBCC	nBCC	nBCC
**6**	sBCC	sBCC	sBCC	sBCC	sBCC	sBCC	sBCC	nBCC
**7**	sBCC	sBCC	sBCC	sBCC	sBCC	sBCC	nBCC	nBCC
**8**	sBCC	sBCC	sBCC	sBCC	sBCC	sBCC	sBCC	nBCC
**9**	sBCC	sBCC	sBCC	sBCC	sBCC	nBCC	nBCC	nBCC
**10**	sBCC	sBCC	sBCC	sBCC	nBCC	nBCC	nBCC	nBCC
**11**	sBCC	sBCC	sBCC	sBCC	nBCC	nBCC	nBCC	nBCC
**12**	sBCC	sBCC	sBCC	sBCC	sBCC	nBCC	nBCC	nBCC
13*	sBCC	sBCC	sBCC	sBCC	nBCC	sBCC	sBCC	sBCC
**14**	sBCC	sBCC	sBCC	sBCC	sBCC	nBCC	nBCC	nBCC

NA = No tissue left for this additional sectioning.

### Follow-up excision

Thirteen of the 14 patients with a nBCC in HE levels 5–8 underwent further excision. Of these, BCC components that were found were of combined superficial and nodular histology in 10 patients, solely nodular in 1 patient and of a combined superficial, nodular and infiltrative histology in 2 patients. The nBCC patient that did not undergo excision had a nodular component in level 5 only (patient number 13, Table 3).

Of the 86 patients that had no deeper component in levels 5–8 (and were thus diagnosed with sBCC even after additional sectioning) 26 underwent excision. In 15 of these, sBCC was also seen in the excision, and in 4 patients no residual BCC was seen. In 7 patients a more aggressive BCC subtype was found in the excision specimen; a nodular component in 1 patient and a combined superficial, nodular and infiltrative subtype in 6 patients. The median clinical diameter of the BCC in these 7 patients was 1.1cm (range 0.6–1.7), compared to 0.7cm (range 0.2–4.0) in patients in whom no further excision was performed (p = 0.049).

## Discussion

The current study was undertaken to examine the yield of an extensive 8 step-section method in patients who were initially diagnosed with sBCC compared to a routine performed histologic examination at 4 levels. We found a more aggressive BCC subtype in 14% of the punch biopsies that were taken from lesions suspect for BCC by using a more extensive step-section method with 4 additional HE levels. Localization in the head&neck region was associated with finding a more aggressive BCC subtype in one of the additional step-sections.

Despite that BCC is the most common cancer worldwide, we were only able to identify two other studies that investigated the yield of sectioning additional levels of BCC biopsy specimens [9,10]. Nguyen et al. compared the sectioning of 3-mm punch biopsy specimens of sBCCs, diagnosed at 1 HE level sectioned at 1020μm to that of 4 additional levels at an interval of 200μm [9]. After every 200μm, 10 sections of 4μm were cut, of which the middle 2 sections were HE stained and evaluated. Thus, the additional 4 HE levels were reviewed at 1260μm, 1500μm, 1740μm and 1980μm, respectively. A total of 116 patients, diagnosed with sBCC based on 1 HE level were evaluated. The BCC was located on the head&neck in 15/116 patients (12.9%), similar to that in our population (11.0%). The authors found that in 26 (22.4%) of the 116 patients that were initially diagnosed with sBCC, a more aggressive BCC subtype was found in one of the additional 4 HE levels. In three of the 26 patients, the more aggressive BCC subtype was found only in the fourth additional level (thus, at 1980μm). The majority of patients in whom a more aggressive BCC subtype was found was diagnosed with nBCC (n = 16, 62.5%), followed by nodular/micronodular (n = 4), nodular/micronodular/infiltrative (n = 3), 1 iBCC, 1 mBCC and 1 nBCC/iBCC. The biopsy specimens that we evaluated were standard sectioned up to 1300μm, and the additional 4 levels started at 1500μm. One of the 26 patients that Nguyen et al. evaluated had a more aggressive BCC subtype that was only found in the first additional level (thus, at 1260μm). This patient might not have been included using our sectioning protocol, because we only included patients with sBCC up to a depth of 1400μm. Van Delft et al. retrospectively assessed 85 patients diagnosed with BCC, and evaluated the proportion of a more aggressive BCC subtype that was missed by evaluation on 1 or 2 levels, using 4-level diagnosis as reference standard. They found a more aggressive BCC subtype in 14 patients (16.5%). However, it has to be noted that this included the shift from a nBCC to iBCC subtype in 5 patients, which will not always have clinical consequences. Further, the authors did not mention the localization of the biopsy specimens. It is challenging to compare their results to ours, because of the retrospective design of their study and the fact that the authors did not mention the depth at which the biopsy specimens were evaluated [10].

The current results also show that 7 patients with sBCC in 8-level biopsy sectioning still had a more aggressive subtype in their excision specimen. This might be caused by sampling error, because a punch biopsy is often 2-3mm in diameter and thus reflects only a sample of the total lesion. This can be especially challenging in larger lesions, which might be true for the current 7 patients as well. The median clinical diameter of the BCC in these 7 patients was 1.1cm (range 0.6–1.7), compared to 0.7cm (range 0.2–4.0) in patients in whom no further excision was performed. Although this was not significant (p = 0.049), which might be caused by the small sample size, a difference of 5mm might be considered quite substantial.

As diagnosing BCCs constitutes a large part of the daily routine of dermatopathologists, one cannot overlook the additional time and costs that go hand-in-hand with additional step-sectioning. It took pathology technicians in the current study 10–15 minutes extra time to section 4 additional HE levels. Costs were estimated at 15–20 euros, which included laboratory consumables. In our opinion, these extra costs do not outweigh the consequences of misdiagnosing 14% of patients as sBCC, as it is likely that recurrences (along with their additional costs) will occur more often when more aggressive BCC subtypes are not treated appropriately [11].

Strengths of the current study include its large sample size and the use of a thorough step-section method. Limitations include the fact that we did not analyze the cost-effectiveness of sectioning 4 additional levels to 4 standard levels. Another limitation is that the clinical tumor diameter was available in only 56 patients. A larger tumor diameter might lead to an increased risk of sampling error when a biopsy is taken. A final limitation is that although in the majority of patients a 2- or 3mm punch biopsy was performed, a 4- or 5-mm punch biopsy was used in 5 patients (5%). Consequently, relatively less tissue of these 5 patients was evaluated. Of the 14 patients with a more aggressive BCC subtype in levels 5–8, only 1 underwent a 4-mm punch biopsy and all others a 2- or 3-mm. All 7 patients with sBCC in 8-level biopsy sectioning, but a more aggressive subtype in their excision specimen, underwent a 2- or 3-mm punch biopsy.

### Conclusions

More extensive sectioning of sBCC biopsy specimens, especially in the head&neck area, leads to a more accurate BCC subtype diagnosis and has a relevant impact on clinical management. Further studies are needed to weigh the advantages of more extensive sectioning of sBCC biopsy specimens against its cost.

## Supporting information

S1 Fig Schematic overview of the histopathological examination process of a punch biopsy specimen suspicious for basal cell carcinoma. (TIF)

S1 File (XLSX)
