# Supplementary figures and images for: Superficial basal cell carcinoma, think deeper: Step sectioning of skin biopsy specimens yields 14% more aggressive subtypes
Source: PLoS One. 2022 Jan 20;17(1):e0256149. doi: 10.1371/journal.pone.0256149 (PMC8775292; doi:10.1371/journal.pone.0256149)

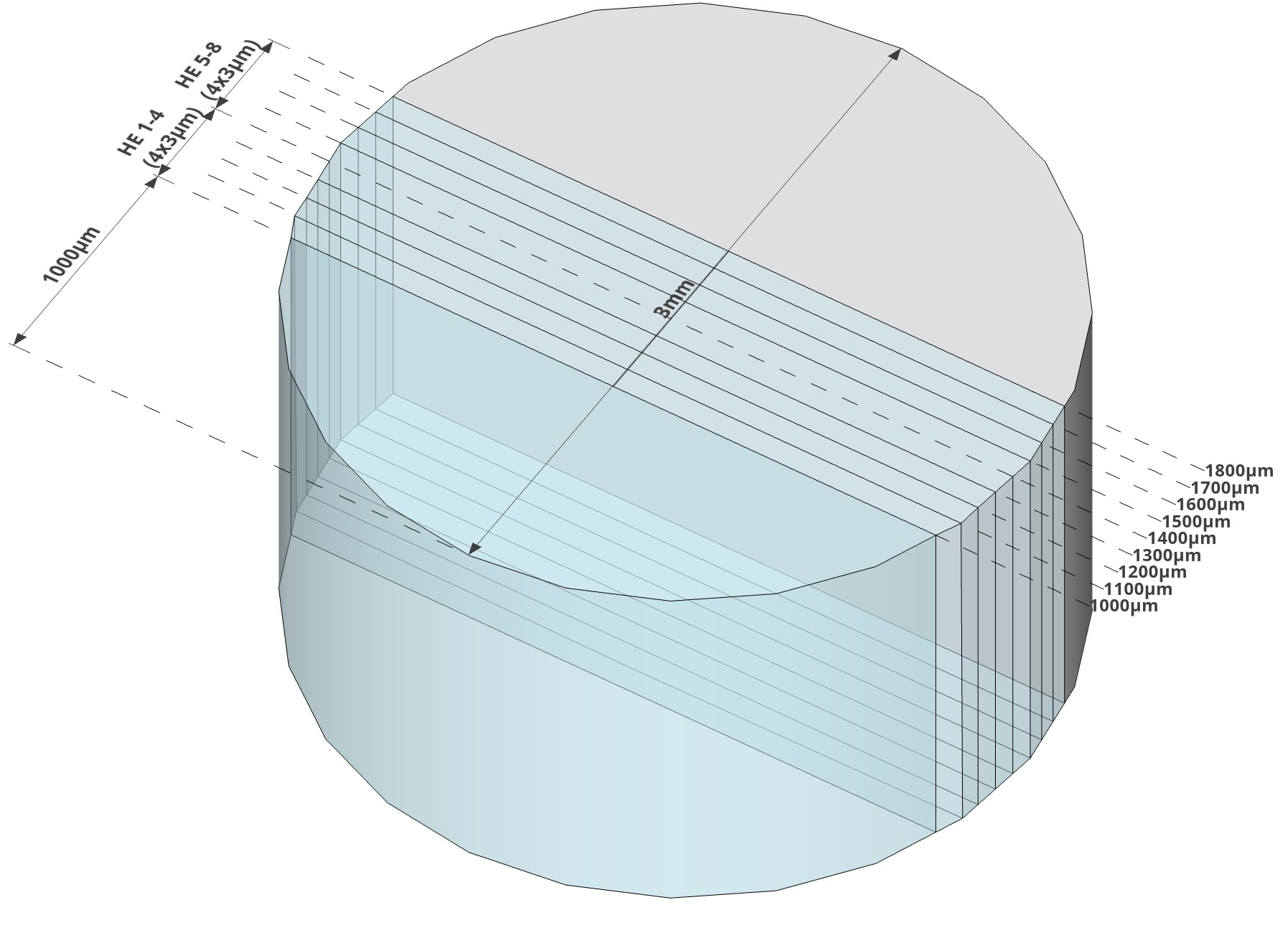

Supplement: S1 Fig — (TIF) [file pone.0256149.s001.tif]
